# Phenolic Glucosides from *Dendrobium aurantiacum* var. *denneanum* and Their Bioactivities

**DOI:** 10.3390/molecules18066153

**Published:** 2013-05-23

**Authors:** Liang Xiong, Zhi-Xing Cao, Cheng Peng, Xiao-Hong Li, Xiao-Fang Xie, Ting-Mo Zhang, Qin-Mei Zhou, Lian Yang, Li Guo

**Affiliations:** 1 State Key Laboratory Breeding Base of Systematic Research, Development and Utilization of Chinese Medicine Resources, Sichuan Province and Ministry of Science and Technology, Chengdu 610075, Sichuan, China; E-Mails: xiling0505@126.com (L.X.); caozhixing007@163.com (Z-X.C.); xxf14544@163.com (X.-F.X.); 2 Pharmacy College, Chengdu University of Traditional Chinese Medicine, Chengdu 610075, Sichuan, China; E-Mails: lixiaohong136@126.com (X.-H.L.); zhangtmchengdu@163.com (T.-M.Z.); zhqmyx@sina.com (Q.-M.Z.); yanglian080910@163.com (L.Y.); 3 Sichuan Wan’an Dendrobe Industry Development Co., Ltd., Chengdu 610072, Sichuan, China

**Keywords:** *Dendrobium aurantiacum* var.* denneanum*, lignan glucosides, phenylpropanoid glycosides, bioactivities

## Abstract

A new 8,4'-oxyneolignane glucoside **1** has been isolated from the stems of *Dendrobium aurantiacum* var.* denneanum* together with six known phenolic glucosides **2**−**7**. The structure of the new compound, including its absolute configuration, was determined by spectroscopic and chemical methods as (−)-(7*S*,8*R*,7'*E*)-4-hydroxy-3,3',5,5'-tetramethoxy-8,4'-oxyneolign-7'-ene-7,9,9'-triol 7,9'-bis-*O*-*β*-D-glucopyranoside (**1**). In the *in vitro* assays, compound **1** and (−)-syringaresinol-4,4'-bis-*O*-*β*-D-glucopyranoside (**2**) showed evident activity against glutamate-induced neurotoxicity in PC12 cells. Shashenoside I (**4**) showed a selective cytotoxic activity with the IC_50_ value of 4.17 μM against the acute myeloid leukemia cell line MV4-11, while it was inactive against 10 other human tumor cell lines.

## 1. Introduction

*Dendrobium aurantiacum* Rchb.f. var.* denneanum* (Kerr.) Z. H. Tsi (Orchidaceae) is widely distributed and cultivated in southern China, Burma, Laos and South Asia. The stem of the plant, commonly referred to as “Shihu” or “Huangcao” in Chinese, has been used in traditional Chinese medicine for its antipyretic, eyesight improving, immunomodulatory, antioxidant and anti-aging effects [1,2]. Previous chemical investigations on this plant have resulted in more than twenty phenolic secondary metabolites, including bibenzyls, phenanthrenes, fluorenones, phenylpropanoids, flavones and coumarins [3,4,5,6]. In the course of our search for bioactive natural products, the stems of *D. aurantiacum* var.* denneanum* were shown to afford seven phenolic glucosides, including a new 8,4'-oxyneolignane 7-*O*-glucoside **1** and six known phenolic glucosides **2**−**7**. Based on IUPAC recommendations for the nomenclature of lignans and neolignans [7], **1** was identified as (−)-(7*S*,8*R*,7'*E*)-4-hydroxy-3,3',5,5'-tetramethoxy-8,4'-oxyneolign-7'-ene-7,9,9'-triol 7,9'-bis-*O*-*β*-D-glucopyranoside. All the compounds were assessed for their neuroprotective activity against glutamate-induced toxicity in PC12 cells and cytotoxic activity against 11 kinds of human tumor cells. This paper describes the isolation, structure elucidation, and biological assays of these compounds.

## 2. Results and Discussion

**Figure 1:**
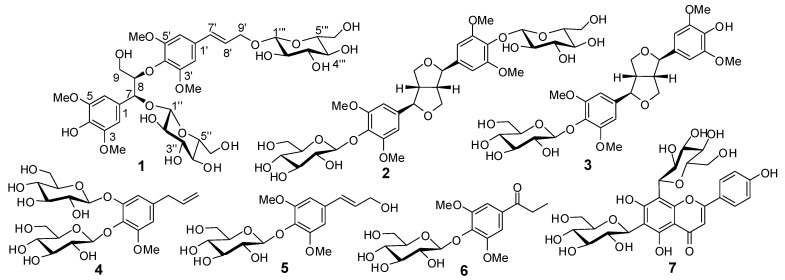
Structures of compounds **1**–**7**.

The EtOH extract of the stem of *D. aurantiacum* var.* denneanum* was suspended in water and successively partitioned with EtOAc and *n*-BuOH. Separation of the *n*-BuOH fraction by column chromatography provided compounds **1**–**7** (Figure 1). The known compounds **2**–**7** were identified as (−)-syringaresinol-4,4'-bis-*O*-*β*-D-glucopyranoside (**2**) [8], syringaresinol-4'-*O*-D-monoglucopyranoside (**3**) [9], shashenoside I (**4**) [10], syringin (**5**) [11], 1-[4-(β-D-glucopyranosyloxy)-3,5-dimethoxyphenyl]-1-propanone (**6**) [12], and vicenin-2 (**7**) [13] by comparing their spectroscopic data with those reported in the corresponding literature.

**Figure 2:**
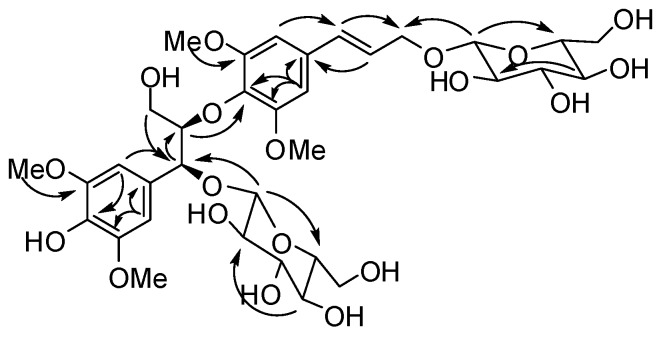
Key HMBC correlations of **1**.

Compound **1** was obtained as a colorless gum, and the presence of OH (3,374 cm^−1^) and aromatic (1,588 and 1,502 cm^−1^) groups were indicated by its IR spectrum. The molecular formula of C_34_H_48_O_19_ was indicated by a HRESIMS peak at *m/z* 783.2681 [M+Na]^+^. The ^1^H-NMR spectrum of **1** (Table 1) showed signals attributable to two symmetrically 1,3,4,5-tetrasubstituted aromatic rings at *δ* 6.77 (H-2', 6') and 6.72 (H-2, 6), together with two six-proton aromatic methoxy singlets at *δ* 3.78 and 3.73. Signals for two olefinic methines and an oxymethylene at *δ* 6.59 (d, *J* = 16.0 Hz, H-7'), 6.35 (dt, *J* = 16.0, 6.0 Hz, H-8'), 4.43 (dd, *J* = 13.0, 6.0 Hz, H-9'a), and 4.20 (dd, *J* = 13.0, 6.0 Hz, H-9'b) suggested the presence of a *trans*-arylpropenoxy unit. In addition, an arylpropanediyloxy unit was indicated by signals of a vicinal coupling system attributed to two oxymethines at *δ* 5.11 (d, *J* = 3.5 Hz, H-7) and 4.27 (H-8), and an oxymethylene at *δ* 3.59 (H-9a) and 3.17 (H-9b). Two diagnostic doublets attributed to anomeric protons at *δ* 4.34 (d, *J* = 7.5 Hz, H-1'') and 4.22 (1H, d, *J* = 8.0 Hz, H-1'''), together with partially overlapped signals assigned to oxymethylene and oxymethine protons between *δ* 3.00 and 3.70, suggested the presence of two *β*-glycopyranosyl units in **1** [14]. The ^13^C-NMR and DEPT spectra of **1** revealed carbon signals corresponding to the above units and quaternary aromatic carbons (Table 1). These spectroscopic features suggested that **1** was a syringylglycerol-8-*O*-4'-sinapyl alcohol ether (SGSE) *β*-diglucopyranoside [15]. Comparison of the ^13^C-NMR data of **1** with those of *erythro*-SGSE showed that the resonances for C-7 and C-9' in **1** were deshielded significantly by Δ*δ* +6.0 and +6.7 ppm, whereas the C-1, C-8, and C-8' resonances were shielded by Δ*δ* −4.5, −2.6, and −3.7 ppm, respectively. This revealed that two *β*-glycopyranosyl units were attached to C-4 and C-9', which was further confirmed by the HMBC correlations of H-1'' with C-4 and H-1''' with C-9' (Figure 2). 

**Table 1.** ^1^H- (500 MHz) and ^13^C-NMR (125 MHz) data of **1 **(in DMSO-*d*_6_, *δ* in ppm, *J* in Hz).

No.	*δ*_H_	*δ*_C_	No.	*δ*_H_	*δ*_C_
1	–	129.8	1''	4.34 d (7.5)	103.1
2	6.72 s	106.1	2''	3.18 m	75.2
3	–	148.2	3''	3.06 m	77.8
4	–	135.6	4''	3.03 m	71.1
5	–	148.2	5''	3.02 m	78.3
6	6.72 s	106.1	6''a	3.66 (overlapped)	62.1
7	5.11 d (3.5)	79.5	6''b	3.41 (overlapped)	–
8	4.27 m	85.0	1'''	4.22 d (8.0)	103.2
9a	3.59 m	61.2	2'''	3.00 m	74.5
9b	3.17 m	–	3'''	3.13 m	77.4
1'	–	133.1	4'''	3.12 m	70.9
2'	6.77 s	104.8	5'''	3.23 m	77.9
3'	–	153.7	6'''a	3.62 (overlapped)	61.9
4'	–	136.7	6'''b	3.40 (overlapped)	
5'	–	153.7	3/5-OMe	3.73 s	57.0
6'	6.77 s	104.8	3'/5'-OMe	3.78 s	57.0
7'	6.59 d (16.0)	132.2			
8'	6.35 dt (16.0, 6.0)	126.8			
9'a	4.43 dd (13.0, 6.0)	69.6			
9'b	4.20 dd (13.0, 6.0)	–			

Enzymatic hydrolysis of **1** produced the aglycone **1a** and a sugar, which was further identified as D-glucose by the positive optical rotation ([α]20D +45.5) [16,17,18] and TLC comparison with an authentic sugar sample. Comparison the ^1^H-NMR and HREIMS data of **1a** with those of known 8,4'-oxyneolignane led characterization of **1a** as *erythro*-SGSE [15]. The CD spectra of **1** and **1a** showed negative Cotton effects at 235 nm (Δ*ε* −0.18) and 239 nm (Δ*ε* −0.22), respectively, indicating an 8*R* configuration for **1** and **1a** [18,19,20]. Thus, compound **1** was determined to be (−)-(7*S*,8*R*,7'*E*)-4-hydroxy-3,3',5,5'-tetramethoxy-8,4'-oxyneolign-7'-ene-7,9,9'-triol 7,9'-bis-*O*-*β*-D-glucopyranoside.

The isolated compounds were assessed for their cytotoxic activity against 11 kinds of human tumor cells, including the lung cancer cell lines H1975, H358 and A549, hepatocellular carcinoma cell lines HepG-2 and SMMC7721, colorectal carcinoma cell line HCT116, mammary carcinoma cell lines MDA-MB-231 and MCF-7, melanoma cell line A2058, pancreatic cancer cell line PANC-1, and acute myeloid leukemia cell line MV4-11 in *in vitro* bioassays. Among them, compound **4** showed a selective cytotoxic activity against acute myeloid leukemia cell line MV4-11, with the IC_50_ value of 4.17 μM. Other compounds were inactive against the tested human tumor cells at the concentration of 10 μM.

In addition, the protective activity of the compounds against glutamate-induced neurotoxicity in PC12 cells was evaluated by an MTT assay [18,21]. The results showed that glutamate induced an inhibition of MTT reduction, while compounds **1** and **2** showed neuroprotective activity at a concentration of 10 μM, with the relative protection of 25.7 ± 2.2% and 19.3 ± 5.6%, respectively (the positive control MK-801, 85.9 ± 3.2%). Thus, lignan glucosides **1** and **2 **may be effective in neurodegenerative disorders.

## 3. Experimental

### 3.1. General

NMR spectra were recorded on a INOVA-500 spectrometer. HRESIMS were measured with Waters Synapt G_2_ HDMS. IR were recorded on a Vector 22 FT-IR spectrometer. UV spectra were obtained on a Shimadzu UV-260 spectrophotometer. Optical rotations were measured with a Perkin-Elmer 341 plus. CD spectra were recorded on a JASCO J-815 CD spectrometer. Column chromatography was performed with silica gel (200–300 mesh, Yantai Institute of Chemical Technology, Yantai, China) and Sephadex LH-20 (Amersham Pharmacia Biotech AB, Uppsala, Sweden). HPLC separation was performed on an instrument consisting of a Cometro 6000LDS pump and a Cometro 6000PVW UV/VIS detector with an Ultimate (250 × 10 mm) preparative column packed with C_18_ (5 μm). 

### 3.2. Plant Material

The stem of *Dendrobium aurantiacum* var.* denneanum* was collected in April of 2011 from a culture field in Shuangliu, Sichuan Province, China. Plant identity was verified by Prof. Min Li (Chengdu University of TCM, Sichuan, China). A voucher specimen (SSF-20110410) was deposited at the School of Pharmacy, Chengdu University of TCM, Chengdu, China.

### 3.3. Extraction and Isolation

The air-dried stem of *D. aurantiacum* var.* denneanum* (10 kg) was powdered and extracted three times with 95% EtOH (30 L) for 3 h under reflux. The EtOH extract was evaporated under reduced pressure to yield a dark brown residue (530 g), which was suspended in H_2_O (2.5 L) and then successively partitioned with EtOAc and *n*-BuOH (6 × 2.5 L). The *n*-BuOH extract (110 g) was applied to a D-101 macroporous adsorbent resin (1.5 Kg) column. Successive elution of the column with H_2_O, 10% EtOH, 30% EtOH, 50% EtOH, and 95% EtOH (4 L each) yielded five portions. The portion (48 g) eluted by 30% EtOH was separated by MPLC over reversed-phase silica gel eluting with a gradient of increasing MeOH (5−90%) in H_2_O to give eight fractions (A-H). Fraction **7** (185 mg) was precipitated from the fraction D in MeOH. Subsequent separation of fraction F (6.5 g) over Sephadex LH-20 eluted with MeOH−H_2_O (1:1) gave five subfractions (F_1_−F_5_). Subfraction F_2_ was further fractionated via silica gel CC, eluting with CHCl_3_−MeOH (8:1), to yield six fractions (F_2-1_−F_2-__6_). Separation of F_2-2_ with RP semipreparative HPLC (37% MeOH in H_2_O) yielded **1** (6.5 mg) and **2** (38.0 mg). F_2-5_ was further separated by Sephadex LH−20 (MeOH−H_2_O, 3:7), and then purified by RP semipreparative HPLC (34% MeOH in H_2_O), to yield **4** (31 mg). Subfraction F_4_ was fractioned by silica gel CC, eluting with a gradient of increasing MeOH (5−50%) in CHCl_3_, to afford seven fractions (F_4-1_−F_4-7_). F_4-3_ was purified by RP preparative HPLC (35% MeOH in H_2_O) to afford **3** (9 mg), **5** (36 mg), and **6** (11 mg).

*(−)-(7S,8R,7'E)-4-hydroxy-3,3',5,5'-tetramethoxy-8,4'-oxyneolign-7'-ene-7,9,9'-triol* *7,9'-bis-O-β-D glucopyranoside* (**1**): White gum, 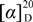 = −12.2 (*c* = 0.20, MeOH); IR (KBr) νmax: 3374, 2920, 1588, 1502, 1462, 1420, 1331, 1226, 1124, 1071, 1023, 835, 706, 615 cm^−1^; UV (MeOH) *λ*_max_ (log *ε*): 204 (4.35), 226 (4.03, sh), 271 (3.67) nm; CD (MeOH): 222 (Δ*ε* +0.16), 235 (Δ*ε* −0.18), 251 (Δ*ε* +0.03), 266 (Δ*ε* −0.12), 290 (Δ*ε* +0.23) nm; ESI-MS *m/z*783 [M+Na]^+^; HRESI-MS: *m/z* 783.2681 [M+Na]^+^ (calcd for C_34_H_48_O_19_Na, 783.2687); ^1^H- and ^13^C-NMR data see Table 1.

### 3.4. Enzymatic Hydrolysis of **1**

A solution of compound **1** (2 mg) in H_2_O (10 mL) was hydrolyzed with *β*-glucosidase (15 mg) at 37 °C for 96 h. The reaction mixture was extracted with EtOAc (3 × 10 mL) to yield the individual EtOAc extract and H_2_O phase after removing the solvents. The aqueous phases were subjected to preparative TLC eluted with MeCN−H_2_O (8:1) to yield the sole sugar, which could be identified as D-glucose by the sign of its positive optical rotation. The EtOAc extracts were purified by preparative TLC using CHCl_3_-MeOH (12:1) to afford **1a** (0.2 mg): 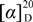 = +13.2 (*c* = 0.02, MeOH); CD (MeOH) 221 (Δ*ε* +1.25), 239 (Δ*ε* −0.22), 270 (Δ*ε* +0.43) nm; ^1^H-NMR (CD_3_OD, 500 MHz) *δ*: 6.76 (2H, s, H-2', 6'), 6.68 (2H, s, H-2, 6), 6.55 (1H, d, *J* = 16.0 Hz, H-7'), 6.38 (1H, dt, *J* = 16.0, 6.0 Hz, H-8'), 4.99 (1H, d, *J* = 3.5 Hz, H-7), 4.27 (2H, d, *J* = 6.0 Hz, H_2_-9'), 4.19 (1H, m, H-8), 3.88 and 3.83 (each 6H, s, OMe-3, 5, 3', 5'), 3.87 (1H, m, H-9a), 3.48 (1H, m, H-9b); ESI-MS * m*/*z*: 459 [M+Na]^+^; HR-ESI-MS* m*/*z*: 459.1624 [M+Na]^+^ (calcd for C_22_H_28_O_9_Na, 459.1631).

### 3.5. Cell Culture and Assessment of Cytotoxic Activity against Human Tumor Cells

The human lung cancer cell lines H1975, H358 and A549, human hepatocellular carcinoma cell lines HepG-2 and SMMC7721, human colorectal carcinoma cell line HCT116, human mammary carcinoma cell lines MDA-MB-231 and MCF-7, human melanoma cell line A2058, human pancreatic cancer cell line PANC-1, human acute myeloid leukemia cell line MV4-11 were obtained from the American Type Culture Collection (ATCC) and grown in RPMI1640, DMEM or IMDM containing 10% fetal bovine serum (v/v) in 5% CO_2_ at 37 °C. Cells (2 × 10^3^–10 × 10^3^) were seeded in 96-well plates and cultured for 24 h, followed by the test compounds treatment at concentrations of 0.625–20 µM for 72 h. After the culture period, 20 *µ*L of MTT (5 mg/mL) was added per well and incubated for 4 h at 37 °C, then 50 μL of 20% acidified SDS was used to lyse the cells. Finally, absorbance was measured at 570 nm using a microplate reader. Each assay was replicated three times. The effect of the compounds on tumor cells viability was calculated and expressed by IC_50_ of each cell line.

### 3.6. Cell Culture and Assessment of Neuroprotective Activity

PC12 cells at a density of 5 × 10^3^ cells per well in 96-well plates were suspended in Dulbecco’s Modified Eagle’s Medium (DMEM, Gibco) media supplemented with 5% fetal bovine serum (FBS, Hyclone) and 5% horse serum, penicillin (100 IU/mL), streptomycin (100 μg/mL), and L-glutamine (2 μM) and incubated in a CO_2_ incubator (5%) at 37 °C for 24 h. The cells were pre-treated with test compounds and MK-801 for 1 h, respectively, and then exposed to glutamate (50 μM). After incubation for an additional 24 h, MTT (0.5 mg/mL) was added to the medium and incubated for 4 h. Absorbance was measured at 570 nm using a microplate reader, the cell viability was evaluated by relative protection, which was calculated as 100 × [optical density (OD) of test compound + glutamate-treated culture − OD of glutamate-treated culture]/[OD of control culture − OD of glutamate-treated culture].

## 4. Conclusions

Several *Dendrobium* species are not only ornamental plants, but are also used as Traditional Chinese Medicines. To search for bioactive natural products from the botanical drugs, we carried out an examination of the *n*-BuOH soluble portion of the ethanolic extract of the stems of *D. aurantiacum* var.* denneanum*. A new 8,4'-oxyneolignane 7-*O*-glucoside, (−)-(7*S*,8*R*,7'*E*)-4-hydroxy-3,3',5,5'-tetramethoxy-8,4'-oxyneolign-7'-ene-7,9,9'-triol 7,9'-bis-*O*-*β*-D-glucopyranoside (**1**) was isolated, together with two 7,9':7',9-diepoxylignan glucosides **2**−**3**, four phenylpropanoid glycosides **4**−**6** and a flavone C-glycoside **7**. The results showed that the types of compounds of *D. aurantiacum* var.* denneanum* were similar to other species of *Dendrobium*, including* D. chrysanthum*, *D. loddigesii*, *D. nobile*, * D. moniliforme*, *D. trigonopus*, and * D. aphyllum*. Previous phytochemical studies of these species have also led to the isolation of lignans [22,23,24,25,26,27], phenylpropanoids [28], and flavonoids [26,27]. However, to fully elucidate the systematic correlation of these *Dendrobium* plants, further phytochemical investigations are necessary. In addition, it deserves to be mentioned that most of the lignans from *Dendrobium* plants were 7,9':7',9-diepoxylignans, while 8,4'-oxyneolignans were rarely reported. To the best of our knowledge, in *Dendrobium* 8,4'-oxyneolignans have been only reported from *D. chrysanthum* so far, which implied chemotaxonomic significance between *D. aurantiacum* var.* denneanum* and *D. chrysanthum*. In the *in vitro* assays, compounds **1** [(−)-(7*S*,8*R*,7'*E*)-4-hydroxy-3,3',5,5'-tetramethoxy-8,4'-oxyneolign-7'-ene-7,9,9'-triol 7,9'-bis-*O*-*β*-D-glucopyranoside] and **2** [(−)-syringaresinol-4,4'-bis-*O*-*β*-D-glucopyranoside] showed neuroprotective activity in PC12 cells, while **4** (shashenoside I) had selective cytotoxic activity against the acute myeloid leukemia cell line MV4-11.
